# Large Fibrovascular Polyp at the Lesser Curvature of the Stomach: A Case Report and Literature Review

**DOI:** 10.7759/cureus.74293

**Published:** 2024-11-23

**Authors:** Huy Q Nguyen, Huy L Phan, Toan K Dang, Hung D Mai, Hien T Tran, Nguyen K Tran Pham

**Affiliations:** 1 Department of General Surgery, People's Hospital 115, Ho Chi Minh, VNM

**Keywords:** fibrovascular, large fibrovascular polyp, lesser curvature, polyps, stomach

## Abstract

Fibrovascular polyps are rare, pedunculated, tumor-like lesions usually found in the esophagus. Their occurrence in the stomach is exceedingly rare. In the literature review, several case reports documented fibrovascular polyps developing in the stomach. In this article, we presented a case of a large fibrovascular polyp at the stomach's lesser curvature, protruding from the lesser curvature forward to the vascular spleen and the tail of the pancreas. The patient was admitted with signs of severe blood loss and underwent splenectomy with a misdiagnosis of splenic rupture at a secondary hospital before. The surgery was performed to remove the fibrovascular polyp with wedge gastric resection and distal pancreatectomy.

## Introduction

Fibrovascular polyps (FVPs) are uncommon, benign, pedunculated lesions resembling submucosal tumors. These lesions consist of fibrous tissue, adipose tissue, and vascular elements [1-3]. Typically located in the esophagus, hypopharynx, or, exceptionally, the colon, FVPs originating from the stomach are exceedingly rare, with only a few documented cases in the medical literature [3,4]. Smaller polyps may not cause any symptoms. Then, the symptoms of esophageal FVPs are usually evident if the tumor develops (dysphagia, substernal discomfort, and asphyxiation), even gastrointestinal bleeding caused by ulcerated FVPs [1,3]. Therefore, we report a case of FVP in the lesser curvature of the stomach that was detected and removed through surgery.

## Case presentation

A 27-year-old male patient presented to the local hospital on the first day of illness with the chief complaints of left hypochondrium abdominal pain and non-bloody vomiting. Family history and past medical history were uneventful. There is no history of trauma or previous impact, and no signs of external skin injury were noted. Upon general examination, the body temperature was 37°C, heart rate was 130 beats per minute, and blood pressure was 100/55 mmHg. The physical examination revealed pale conjunctiva and abdominal pain when under pressure. Laboratory findings demonstrated a hemoglobin level of 10 g/dL, an INR (international normalized ratio) of 2.4, and an aPTT (activated partial thromboplastin time) of 57 seconds. The patient was diagnosed with hemorrhagic shock due to splenic rupture-coagulopathy and subsequently underwent spleen resection surgery at the district hospital. After one day, the patient was transferred to our hospital in a state of intubation, requiring vasopressors, with a heart rate of 110 beats per minute, blood pressure of 140/90 mmHg, body temperature at 37°C, and respiratory rate of 18 breaths per minute.

Upon clinical examination, the patient remained awake, responsive to painful stimuli, and had pale conjunctiva. Blood test results revealed a hemoglobin concentration of 10.9 g/dL, HCT (hematocrit) of 33%, WBC (white blood cell) count of 40k/uL, and NEU (neutrophils) of 87.5% (Table 1). An abdominal ultrasound showed minimal fluid in the splenic fossa. Other results were within normal limits. Continuous Douglas drainage tube monitoring and blood tests every eight hours were initiated. After 24 hours, approximately 1 L of blood was drained from the tube, with a constant decline in hemoglobin, reaching a nadir of 6.7 g/dL. Despite continuous blood transfusions, the hemoglobin levels did not improve. Therefore, the urgency of surgical exploration was decided upon to determine the underlying cause.

**Table 1:** Laboratory values at the time of admission WBC: white blood cell, INR: international normalized ratio, aPTT: activated partial thromboplastin time

Lab	Value	Reference Range
WBC count	40	4.0-10.0 K/µL
Neutrophil count	87.5%	44-66%
Hemoglobin	10.9	2.2-15.4 g/dL
Hematocrit	33%	38-54%
INR	2.4	1.14-1.27 seconds
aPTT	57	-

**Figure 1:**
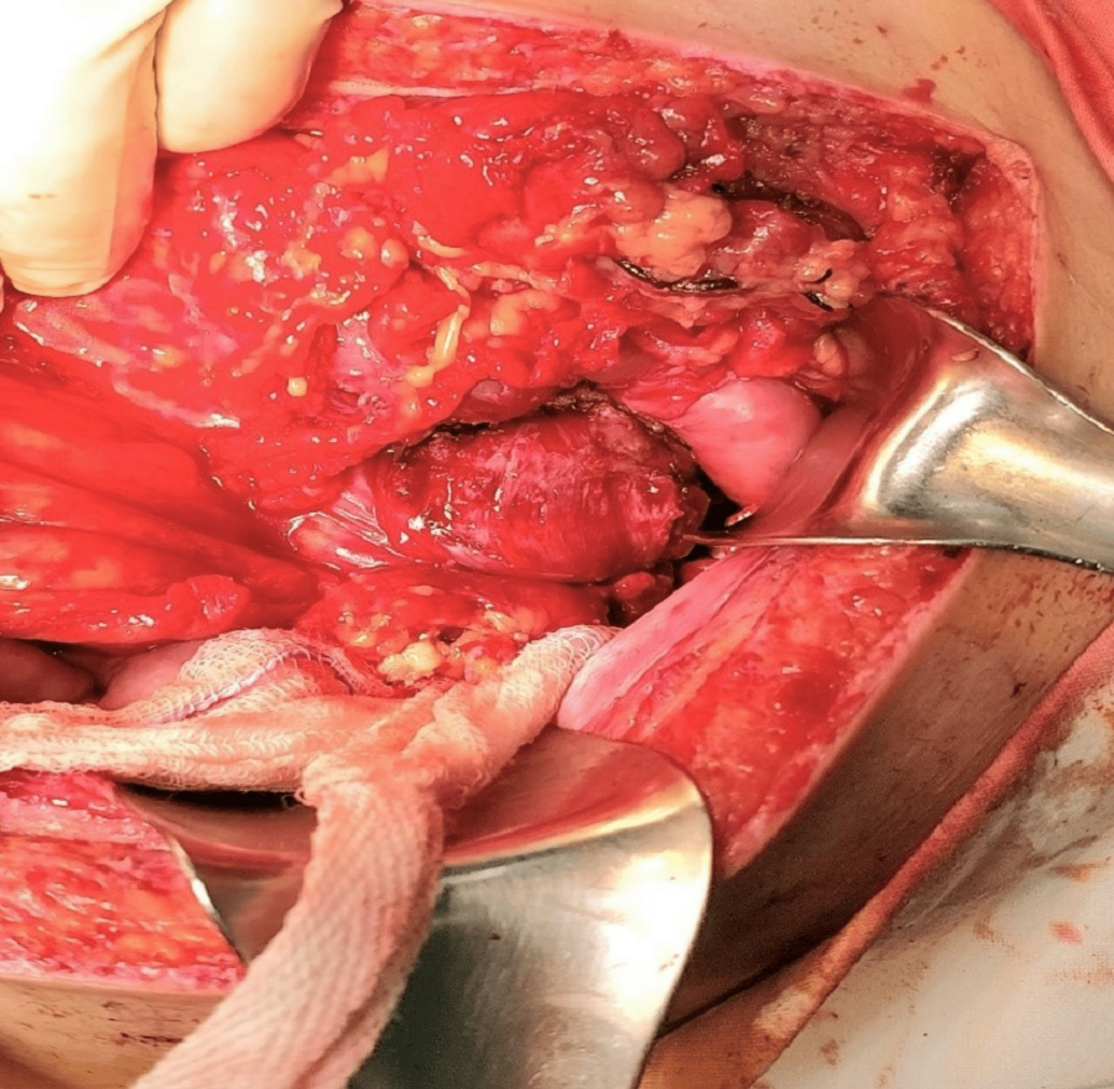
The large fibrovascular polyp at the lesser curvature of the stomach protruding from the lesser curvature forward to the vascular spleen and the tail of the pancreas

During surgery, we identified a large mass approximately 3 × 4 cm in size, with a stalk originating from the upper third of the lesser curvature of the stomach and invading the tail of the pancreas. This mass was bleeding continuously (Figure 1). We decided to perform a wedge gastrectomy and distal pancreatectomy. Postoperatively, the patient was monitored for one week and recovered uneventfully.

**Figure 2:**
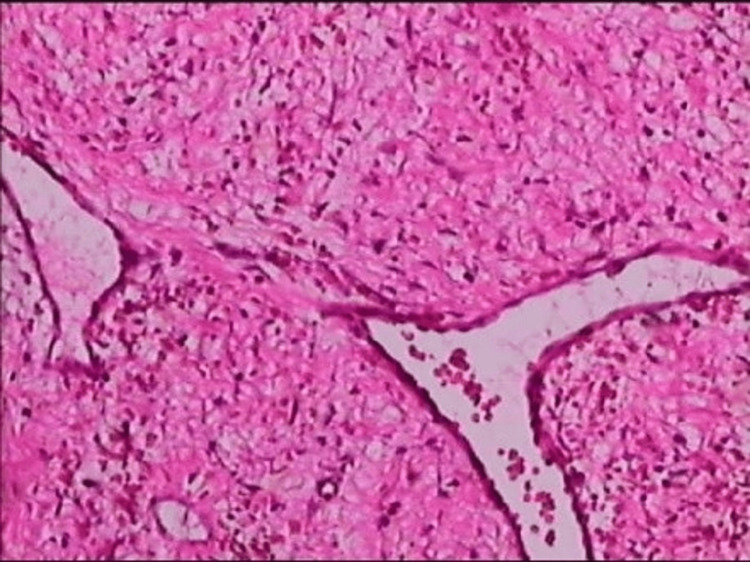
Pathologic findings: irregular blood vessels

**Figure 3:**
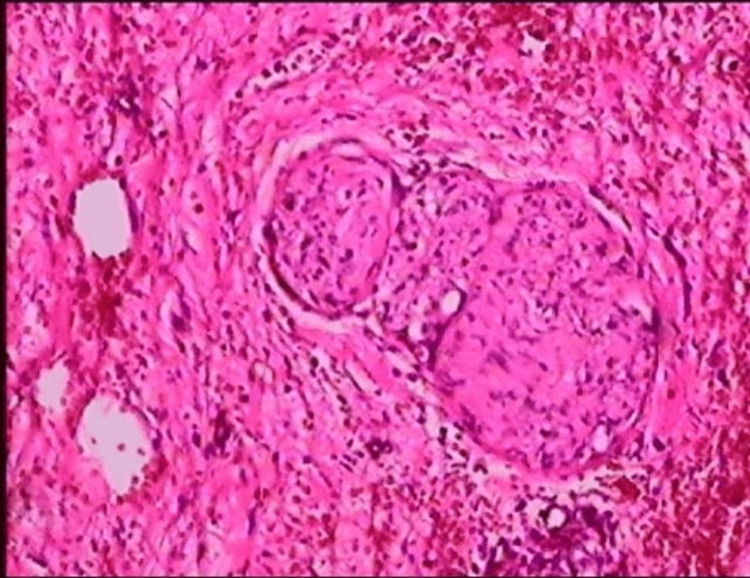
Pathologic findings: increased nerve proliferation

**Figure 4:**
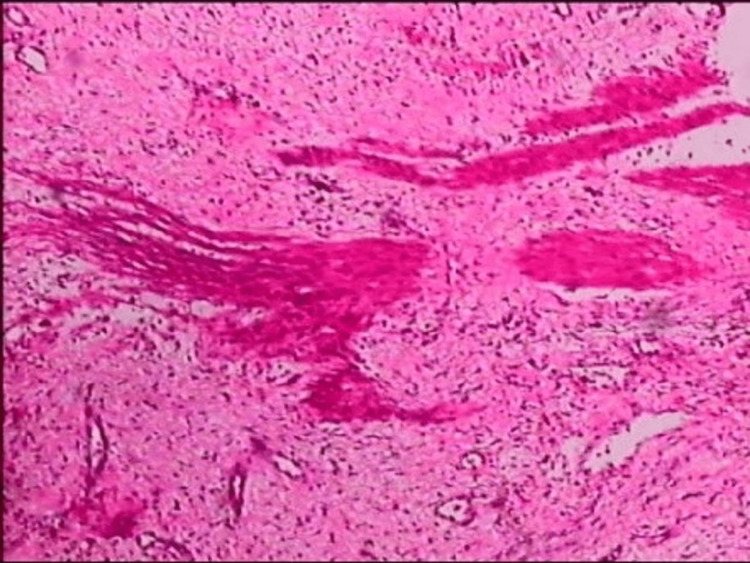
Pathologic findings: smooth muscle tissues

Pathological examination results were consistent with an FVP of the stomach. Microscope findings showed that the submucosal tissue was composed of many irregular blood vessels, smooth muscle tissues, and an increased proliferation of nerves (Figures 2-4).

## Discussion

FVPs are rare benign tumors that typically arise in the proximal part of the esophagus and stomach [1,5]. These tumors are benign and often have a stalk attaching them to the esophagus or stomach [4]. FVPs can present as large masses, especially those with long stalks protruding into the gastric lumen. Common symptoms include dysphagia, substernal discomfort, the sensation of a foreign body in the throat, and occasionally bleeding from ulceration at the polyp's tip [4,5].

The diagnosis of FVP can be achieved through a combination of clinical history and several diagnostic tests, such as barium swallow studies, endoscopy, endoscopic ultrasound, computed tomography (CT), and magnetic resonance imaging (MRI). Imaging studies such as CT scans and MRI are considered the gold standard for determining the nature and origin of the mass [5].

In this case, which was unique, the patient was admitted to the hospital with massive, severe internal bleeding. The patient was misdiagnosed as having a splenic rupture and had a splenectomy at a secondary hospital. Then, due to continued intra-abdominal bleeding, the patient was transferred to our hospital. Through monitoring, we determined that the excessive bleeding persisted, prompting us to do a second surgical procedure to stop the bleeding. During surgery, it was discovered that FVPs of the stomach invaded the tail of the pancreas.

The differential diagnoses of FVP include other lipomatous masses involving the stomach, such as lipomas, angiolipomas, and liposarcomas [6,7]. FVP's histology consists of a combination of fibrous tissue, adipose tissue, and vascular structures, with the proportions of these three components varying depending on the lesion and the area within the same lesion. Therefore, histopathological features are critical to the differential diagnosis [8].

Surgical removal is the preferred treatment for FVPs, especially when they have complications such as recurrent bleeding or obstruction of the digestive and respiratory tract. Endoscopy is typically employed for small polyps with a visible stalk, which can be removed using tools such as a snare, stapler, ligature, laser, or ultrasonic technology. For larger lesions exceeding 10 cm, endoscopic treatment may be challenging, and an open surgical approach is often preferred [2,9,10].

FVPs of the stomach are rare, and due to broad clinical behavior, they can easily be misdiagnosed. These polyps can be mobile within the stomach, making it challenging to distinguish them from other tumors, such as lipomas, angiolipomas, or liposarcomas, using imaging studies such as ultrasound and CT [4,5]. Thus, all surgeons are required to have in-depth knowledge of gastrointestinal pathology. The presence of FVPs in the stomach warrants vigilance and accurate diagnosis to determine the most appropriate treatment approach for each case.

After the surgery, the patient received aggressive treatment, including eight units of 350 mL packed red blood cells and six units of PFC. The patient was given antibiotics, pain relievers, and treatment for water and electrolyte imbalance. Vasopressors were used until the third day after the surgery and monitored continuously until the seventh day postoperatively. The patient recovered and was discharged from the hospital on the 10th day after the surgery. Pathology results, including immunohistochemistry, all confirmed FVPs.

In summary, we reported a case of a large FVP located at the lesser curvature of the stomach, causing continuous bleeding, which was successfully managed with surgical wedge gastric resection.

## Conclusions

Large fibrovascular disease, which occurs in the lesser curvature of the stomach, is not a common condition in clinical practices that results in intra-abdominal bleeding. This disease can be diagnosed with imaging studies such as CT scans. Open or laparoscopic surgery can handle it, and postoperative pathological findings are crucial in determining the diagnosis.
